# Applications of Photodynamic Therapy in Endometrial Diseases

**DOI:** 10.3390/bioengineering9050226

**Published:** 2022-05-23

**Authors:** Gabriela Correia-Barros, Beatriz Serambeque, Maria João Carvalho, Carlos Miguel Marto, Marta Pineiro, Teresa M. V. D. Pinho e Melo, Maria Filomena Botelho, Mafalda Laranjo

**Affiliations:** 1 Institute of Biophysics and Institute for Clinical and Biomedical Research (iCBR), Area of Environment Genetics and Oncobiology (CIMAGO), Faculty of Medicine, University of Coimbra, 3000-548 Coimbra, Portugal; gcorreiabarros@gmail.com (G.C.-B.); beatrizprazserambeque@gmail.com (B.S.); mariajoaosflcarvalho@gmail.com (M.J.C.); cmiguel.marto@uc.pt (C.M.M.); mfbotelho@fmed.uc.pt (M.F.B.); 2 Centre for Innovative Biomedicine and Biotechnology (CIBB), University of Coimbra, 3000-548 Coimbra, Portugal; 3 Clinical Academic Center of Coimbra (CACC), 3004-561 Coimbra, Portugal; 4 Gynecology Service, Coimbra Hospital and University Centre, 3004-561 Coimbra, Portugal; 5 Universitary Clinic of Gynecology, Faculty of Medicine, University of Coimbra, 3004-561 Coimbra, Portugal; 6 Institute of Experimental Pathology, Faculty of Medicine, University of Coimbra, 3000-548 Coimbra, Portugal; 7 Coimbra Chemistry Centre (CQC)-Institute of Molecular Sciences (IMS), Department of Chemistry, University of Coimbra, 3004-535 Coimbra, Portugal; mpineiro@qui.uc.pt (M.P.); tmelo@ci.uc.pt (T.M.V.D.P.e.M.)

**Keywords:** endometrial neoplasms, endometrium, models, animal, photochemotherapy, photodynamic therapy, therapeutics

## Abstract

Photodynamic therapy (PDT) is a medical procedure useful for several benign conditions (such as wound healing and infections) and cancer. PDT is minimally invasive, presents few side effects, good scaring, and is able to minimal tissue destruction maintaining organ anatomy and function. Endoscopic access to the uterus puts PDT in the spotlight for endometrial disease treatment. This work systematically reviews the current evidence of PDT’s potential and usefulness in endometrial diseases. Thus, this narrative review focused on PDT applications for endometrial disease, including reports regarding in vitro, ex vivo, animal, and clinical studies. Cell lines and primary samples were used as in vitro models of cancer, adenomyosis and endometrioses, while most animal studies focused the PDT outcomes on endometrial ablation. A few clinical attempts are known using PDT for endometrial ablation and cancer lesions. This review emphasises PDT as a promising field of research. This therapeutic approach has the potential to become an effective conservative treatment method for endometrial benign and malignant lesions. Further investigations with improved photosensitisers are highly expected.

## 1. Introduction

Photodynamic therapy (PDT), or photochemotherapy, is a medical procedure that uses photosensitiser drugs and visible light to ablate unwanted cells, such as cancer cells, to enhance wound healing, obtain an antimicrobial effect, and others. The interaction of a specific wavelength with a photosensitiser determines its activation and elicits the photodynamic reaction [1].

In the European Union, there are photosensitisers approved for choroidal neovascularisation associated with age-related macular degeneration and pathological myopia (Verteporfin), mild to moderate actinic keratosis of the face and scalp (5-ALA), advanced cases of prostate adenocarcinoma (Palediporfin), and head and neck squamous cell carcinoma (Temoporfin) [2]. Besides the current clinical indications, PDT constitutes a dynamic area of research with huge potential to grow and become a valid treatment option for a wide range of diseases.

The outcome of PDT depends on the photosensitiser’s characteristics [3,4]. After administration and time for bioavailability, a period called drug-light interval (DLI), the area to be treated is irradiated with appropriate wavelength light. The photosensitiser in the ground state passes to an excited singlet state and then to an excited triplet state by intersystem crossing. The triplet state photosensitiser oxidises the available substrates in two reaction types. In type I, the triplet state photosensitiser reacts with organic molecules, forming reactive oxygen species (ROS) such as peroxides or superoxide anion. In type II, interaction with oxygen forms singlet oxygen, the most relevant ROS in PDT [5]. Due to high ROS concentrations, PDT kills photoxidized cells, disrupts associated vasculature, and triggers an inflammatory/immune response [6].

Despite the complex biology, PDT presents several advantages. Photocytotoxicity effects are local and selective, as the photodynamic reaction occurs only where irradiation is made. It is a painless and straightforward procedure eligible for ambulatory, which can be combined with other treatment approaches. It can be minimally invasive according to the treated area [5]. However, patients treated with first-generation photosensitisers unveiled cutaneous photosensitivity after photodynamic treatment [7,8]. Nevertheless, PDT is particularly interesting for superficial lesions located externally or at accessible cavities, like the endometrium.

**Figure 1:**
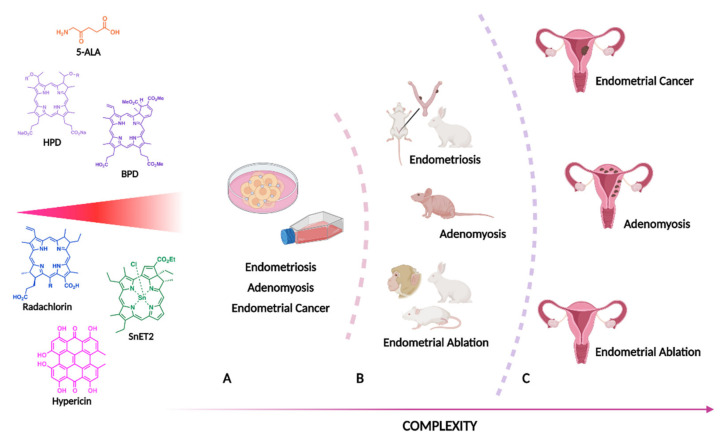
Applications of PDT on endometrial disease encompass in vitro (**A**), animal (**B**) and clinical trials (**C**). Current literature used several photosensitisers, including 5-aminolevulinic acid (5-ALA), hematoporphyrin derivative (**HPD**), benzoporphyrin derivative (**BPD**), radaclorin, tin ethyl etiopurpurin (**SnET2**) and hypericin.

The applicability of PDT for gynaecologic diseases has been addressed in previous reviews. Such works address PDT applications to some endometrial diseases, namely endometrial cancer and endometriosis [9,10]. This work systematically reviews the current evidence of PDT’s potential and usefulness to endometrial disease. Thus, this review includes the reports regarding in vitro, ex vivo, animal, and clinical studies, as schematised in Figure 1.

## 2. Materials and Methods

A narrative review was performed focusing on PDT applications for endometrial disease. A systematic approach was used for the study’s identification and selection, aiming to increase the quality of the manuscript.

The article search was performed in Medline (through PubMed), Cochrane Library, Embase, Web of Science and Clinical Trials databases. The search was performed using the terms Endometrium, Endometrial neoplasms/cancer, Photochemotherapy and Photodynamic Therapy and the boolean operators AND and NOT. Articles published from 1978 onwards were considered, and the last search was performed on April 2022. Only articles published in English and Portuguese were considered. Clinical, animal, ex vivo or in vitro studies evaluating PDT application on endometrial diseases/models were considered.

A standardised approach was used to collect the data, which included the article’s first author, publication year, photosensitiser type, dose, DLI, irradiation characteristics, follow-up period, and main results. The data collected was registered for all studies. The disease type, number and age of patients, histological evaluation, tumour stage, and exam complementary data (ECD) were also noted in the clinical studies. In in vivo studies, the disease type, animal species, model type, procedure or inductor agent, the number of animals, gender, age and weight were also recorded. In the ex vivo studies, sample type or pathology and assays performed were also registered: Finally, for the in vitro studies, the cell line, photosensitiser concentration, number of treatments, and assays performed were recorded.

## 3. Endometrial Pathology

The endometrium is a high turnover tissue influenced by sex hormones that lead to cyclic modifications that allow embryo implantation or culminate in menstruation every month. The basal layer of the endometrium possesses epithelial and mesenchymal stem cells, able to self-renew throughout reproductive life, ensuring a cyclic remodelling of the functional layer [11]. Endometrial cell proliferation is ruled by oestrogens that regulate cell survival and viability as well as mitogenic effects [12]. Also, the dysregulation and dedifferentiation of these phenomena expose endometrial glands to stimuli that play a central role in proliferation and disease, namely hyperplasia and cancer.

Endometrial pathology encompasses benign and malignant lesions. Abnormal uterine bleeding (AUB) is the clinical manifestation of frequent benign pathologies such as adenomyosis and endometrial polyps or dysfunctional conditions. As discussed below, neoplastic diseases often require invasive procedures such as surgery followed by radiotherapy or chemotherapy, which are highly mutilating and compromise the women’s fertility. Endoscopic access to the uterus puts PDT in the spotlight for endometrial disease treatment. PDT is minimally invasive, presents few side effects, good scaring, and is able to minimal tissue destruction maintaining organ anatomy and function. Therefore, PDT has the potential to become an effective conservative treatment method for radical surgical ineligibility and fertility-sparing cases.

### 3.1. Endometrial Cancer

Endometrial neoplasms are the most frequent gynaecological malignancy in developed countries, being the fourth most frequent in women and representing about 6% of all cancers [13]. Almost 80% of endometrial neoplasms are diagnosed at an early stage, but a few are diagnosed in women under 40 years old. Endometrial cancer treatment includes surgical staging that begins with a total hysterectomy and bilateral salpingo-oophorectomy. Lymphadenectomy is an integral part of comprehensive surgical staging, but the utility in the early stage is still controversial [14].

Conservative therapies are necessary for women with significant medical comorbidities, obese women to whom surgery is technically limited, patients with specific tumour characteristics or particular genetic backgrounds and women with childbearing capacity [15,16]. Regrettably, current conservative approaches, which are based on progestogens, have inherent risks: the treatment being ineffective, the risk of relapse, missing a diagnosis of synchronous ovarian lesions and lymph node involvement. Patients aged ≤ 40 years present a higher recurrence rate and worse progression-free survival [17,18]. In fact, there are reports of hysteroscopic resection followed by progestogens with better outcomes considering response rate, recurrence rate and live birth rate [19]. In this context, a continuous search for new diagnostic and therapeutic methods is still required [20].

In vitro studies showing PDT’s potential to treat endometrial cancer were described in 10 papers, detailed in Table 1. Raab and colleagues showed the endometrial cancer cell line HEC1-1A remained unaffected by hematoporphyrin derivative (HPD) up to 10 µg/mL, after both 24 and 48 h of incubation, in the absence of light [21]. Nevertheless, PDT caused cell eradication for the more potent therapies, such as 10 g/mL HPD with a 24 h incubation period or 5 g/mL with a 48 h incubation time, indicating a treatment with limited secondary effects [21]. Later, Schneider-Yin and colleagues studied 5-aminolevulinic acid (5-ALA) and hypericin in the same endometrial adenocarcinoma cell line. 5-ALA based PDT decreased cell survival, but no enhancement was seen with the combination of both photosensitisers after 635 nm irradiation. A slight additive effect was observed when irradiation was made with white light [22].

Combination studies associating PDT with chemotherapy were also performed. The association of HPD and carboplatin led to increased cytotoxicity and ROS production [23]. While hydroxyl radical and superoxide anion concentrations remained stable, and a slight increase of hydrogen peroxide was observed after PDT, ROS concentrations increased when combined with carboplatin. Moreover, PDT increased necrotic cells while PDT combined with carboplatin increased apoptotic and necrotic cells. The viability of cells submitted to PDT depended on the light energy deposited. The association with carboplatin showed better outcomes when low powers (330–660 mJ) were used [23]. Thus, PDT also showed the potential of therapeutic association to reduce undesirable effects of standard treatments by lowering the effective dosage of chemotherapeutics.

Using another endometrial cancer cell line, HEC-1B, hypericin action was further explored [24]. Hypericin uptake remained relatively steady from 3 to 20 h, and sub-cellular fractions showed a tendency for nuclear accumulation. HSP70, P21, and P53 expression synergistically induced photoactivation, while the cell cycle accumulated in the G2/M phase. Poly(ADP-ribose)polymerase (PARP) activation determined apoptotic cell death, while necrosis was observed for higher fluences [24].

Primary cells from ten patients corroborated HPD based PDT outcomes. Decreased proliferation and enhancement of necrosis were observed. Moreover, PDT induced no damage to the basal membrane (laminin) but led to shallow EGFR expressions [25]. 

Radachlorin, a derivative of the well-known water-soluble green pigment chlorophyll α, is a promising PDT photosensitiser and was first introduced as a potential drug by E. Snyder et al. in 1942 [26]. Kim and colleagues showed Radachlorin-PDT IC50 values of 55.4 µM and 20 µM, obtained 24 and 48 h after photosensitising the HEC-1A cells, respectively. Annexin-V-positive and TUNEL-positive cells increased after treatment, mainly after 48 h. PARP and caspase 9 increased when PDT was combined with vascular endothelial growth factor (VEGF) treatment. Also, PDT and PDT + VEGF conditions reduced tubular formation. PDT combined with VEGF suppressed the invasion, reduced prostaglandin-2 production, and reduced EGFR, VEGFR2 and RhoA expression [16].

In clinical practice, the use of PDT in endometrial cancer patients was described in five papers. The first case report, by Dougherty in 1978, described clinical responses of 100 individual tumours, representing ten different types. For the first time, the authors reported a complete response of six endometrial tumours to HPD-PDT in a patient [27]. It took 17 years to publish the following report, where 26 patients with gynaecological cancer were treated, including five endometrial cancer patients. PDT was used as palliative care to eliminate symptoms; however, improvement was temporary. Complete response was achieved in 70.8% of endometrial cancers, and the procedure was considered safe and effective, particularly in locoregional recurrence [28]. Koren and colleagues described the treatment of seven patients with early-stage endometrial carcinoma. The majority of patients had partial response or recurrence in 12 months. The authors pointed to the poor retention of HPD in the target tissue and the influence of microenvironment immune cells. Nevertheless, blood coagulation and carbonisation on the tip of the fibre used in the treatment procedure were observed, which may have influenced dosimetry [29].

During the last two decades, new treatment options have emerged. These methods include hormonal treatments such as levonorgestrel-releasing intrauterine device (LNG-IUS), endometrial ablation, uterine artery embolisation, operative hysteroscopy, sentinel ganglion lymphadenectomy, pharmaceutical treatment and chemo and radiotherapy [33,34]. In a conservative approach, the association of hormonal therapy (HT) and hysteroscopy surgery demonstrated the highest success rate [35]. Continuous endometrial exposure to oestrogens increases the risk of endometrial hyperplasia and cancer, but adding progestogen has a protective role. However, there is a lack of research on HT regarding endometrial cancer [36]. Furthermore, no consensus has been achieved on the best progestogen, dosage, administration method, therapy duration, and the follow-up protocol [37]. On the other hand, the hysteroscopic treatment data is limited to a few case series and reports, where indications, treatment techniques, and follow-up varied substantially [37]. Some clinic methods, such as hysteroscopy followed by progesterone medication, have arisen to fill the therapy gap, but only for early endometrial cancer/atypical hyperplasia [37].

PDT might be a viable option, especially given its lack of mutagenic properties [38]. Besides that, the selectivity to cancer cells can be an essential strategy in lowering the rate of metastasis and relapse of disease and reducing secondary effects [39].

Choi et al. evaluated the effectiveness of Photogem^®^ (a commercial formulation of HDP) as a conservative fertility-sparing treatment in 16 women younger than 35 years old, with early-stage endometrial carcinoma [40] and in one case of low-grade endometrial sarcoma [41]. Photodynamic therapy was used in 11 patients as primary treatment and five patients as a secondary treatment for recurrence after primary hormonal therapy. Complete remission was observed in 12 (75%) out of 16 patients, of which four recurred (33%), results comparable to hormonal therapy and more quickly achieved. Nevertheless, after the second course of PDT, one of the patients with recurrence and one of the non-responders showed complete remission. In this study, 57% of the women with reproductive desire had successful pregnancies, mainly using assisted reproductive techniques [40]. The patient with endometrial sarcoma pointed out an interesting approach to conservative treatment. The 31-year-old patient was submitted to laparoscopic lymphadenectomy, polypectomy, endocervical curettage, and HPD-PDT (endometrium and cervix). PDT was followed by letrozole for six months. No recurrence occurred until 99 months, and in vitro fertilisation resulted in a twin pregnancy [41]. These studies emphasise the safety of PDT regarding reproductive outcomes.

### 3.2. Endometriosis

Endometriosis manifests with chronic pain and infertility. This condition affects roughly 10% (190 million) of reproductive age women and girls globally [42]. Having complex pathophysiology, it is mainly associated with ectopic endometrial glands and stroma outside the uterine cavity, more frequently on the ovary and peritoneum [43], which induces a chronic inflammatory reaction [44]. The anatomical location of endometriotic foci might render PDT challenging to perform. Nevertheless, endometriosis is among the endometrial diseases investigated regarding PDT effectiveness. 

Pharmaceutic, hormonal, and surgery comprise this gynaecological condition’s standard treatment. These treatments alleviate endometriosis symptoms, reduce endometrial lesion size and cell proliferation, and minimise discomfort [45]. Most treatments appear to be more beneficial in stopping disease progression than overcoming this condition. In refractory cases, the option is the complete surgical removal of the affected tissues.

While from the clinical point of view, PDT might seem challenging to apply in endometriotic disease, a series of in vitro and in vivo studies, detailed in Table 1 and Table 2, have been conducted. In fact, primary endometriotic cells showed a higher accumulation of PpIX two hours after incubation, and the laser exposition resulted in strong induction of apoptosis after 24 h [20]. Additionally, endometriotic cells were significantly more responsive to PDT than normal endometrium [30]. Cell growth inhibition was potentiated by associating PDT with verapamil, a PGP inhibitor [30]. Moreover, PpIX accumulation increased after hormonal stimulation [31]. These results pointed to the modulation of PGP and hormonal stimulation as potentially valuable for designing effective phototherapeutic procedures to treat early endometriosis forms [30,31].

Regarding in vivo studies, three reports evaluated 5-ALA and HPD in rabbit and rat animal models [46,47,48]. The endometriosis animal model obtained from Sprague-Dawley rats was achieved by peritoneal autotransplantation of endometrial tissue. 5-ALA based PDT resulted in the endometrial ablation of all explants, with peritoneal necrosis recovery after 16 days and absence of regrowth after three weeks [48]. Based on these results, it was concluded that systemic ALA followed by light exposure at relatively low power densities for periods as brief as 10 min resulted in ablation of endometriotic explants. Thus, it was concluded that systemic ALA activated with low power light for short periods can promote explants ablation [48].

New Zealand white rabbits were used to test HPD-PDT. A similar model of peritoneal transplantation of endometrial tissue was used. In 81% and 60% of the endometrial transplants treated with 100 J/cm^2^ and 50 J/cm^2^ complete or nearly complete endometrial epithelial destruction was achieved, respectively [47]. Later on, a similar approach using a five-fold photosensitiser dose was investigated [46]. The goal was to examine the uptake by endometrial implants in the rabbit peritoneal cavity, assess the extent of HPD binding to other intraperitoneal tissue and organs, and the role of the vapour laser on the endometrial implants. Endometriotic lesions showed higher HPD fluorescence, and PDT led to tissue damage, namely necrosis and hyperaemia, preserving the surrounding tissues [46].

A similar endometriosis rat model was used to study the conversion of 5-ALA into the protoporphyrin IX and biodistribution. Both intravenous and oral administration of 200 mg/kg 5-ALA generated fluorescence in the endometrial implants. High fluorescence was detected in the skin, bladder and uterus, being prominent when the photosensitiser was administered intravenously. Low fluorescence was observed in the peritoneum, bowel mesentery, and eye and no fluorescence was detected in skeletal muscle. The fluorescence was limited to the endometrium when 5-ALA was administered via the intrauterine route. Fluorescence intensity was dependent on the 5-ALA dose and the administration route. The protoporphyrin IX fluorescence peak was achieved 3 to 4 h after therapy, with higher intensity in the implants [49].

### 3.3. Adenomyosis

Adenomyosis is associated with ectopic endometrial glands and stroma surrounded by the hypertrophic and hyperplastic myometrium, leading to enlargement of the uterus and adenomyotic tumour formation [50]. Adenomyosis might be associated with heavy menstrual bleeding, pelvic pain and discomfort [51]. The incidence of adenomyosis is challenging to establish. Nevertheless, it is estimated to affect 2 in 10 women before the age of 40 and 8 in 10 women between 40 and 50 years old [50].

Suzuki-Kakisaka and colleagues cultured primary cells from adenomyosis and endometrium specimens. Adenomyosis cells were more susceptible to 5-ALA-PDT than stromal cells [52]. Loss of cell viability depended on the 5-ALA incubation time and irradiation fluency [52]. These results led the authors to move to a higher complexity model. Immunocompromised mice were ovariectomised, administered with an oestradiol transdermal patch, and transplanted with human adenomyosis tissues [32]. The highest fluorescence was obtained in adenomyosis than in myometrium tissues, up to 3 h after photosensitiser administration. After 5-ALA-PDT, histological studies showed a reduction of epithelial and stromal cells in adenomyosis tissues, more significant in stromal cells, with the absence of necrosis [32].

Considering clinical data, there is a report of a 35-year-old patient with an atypical polypoid adenomyoma submitted to 5-ALA based photodynamic diagnosis. Three hours before intervention, 5-ALA was administered orally (20 mg/Kg). The hysteroscopy allowed the identification of a white protruding lesion in the anterior wall of the lower uterine segment, which was removed with margins. There was no recurrence after six months of follow up [65].

The conservative treatment strategy for adenomyosis includes a surgical approach, namely adenomyomectomy through laparotomy or laparoscopic surgery. However, these therapeutic modalities carry a high risk of uterine rupture during pregnancy [66]. The susceptibility of adenomyosis cells and the increased uptake of 5-ALA in adenomyosis tissues than in the surroundings indicate that 5-ALA may be a suitable photosensitiser. Despite the limited evidence, photodiagnosis with 5-ALA also seemed effective in patients with adenomyosis, revealing no recurrence.

### 3.4. Endometrial Ablation

Endometrial ablation is a minimally invasive procedure performed by hysteroscopy that destroys the active layer of the endometrium. Ablation prevents regrowth and suppresses menstruation and is an option in AUB patients who have met their desired parity. Usually, the candidates are women, with cancer excluded, who present functional or even iatrogenic bleeding and should avoid surgical risks of a hysterectomy due to comorbidities [67]. Limited evidence supports treatment recommendations [68].

PDT-induced endometrial ablation was reported in twelve animal studies, where New Zealand white rabbits and Sprague-Dawley rats were the most used models, as described in Table 2 [53,54,55,56,57,58,59,60,61,62,63,69]. In these studies, photosensitiser bioavailability was evaluated by fluorescence levels in the endometrial and myometrial tissues [55,56,57,58]. HPD is diffusely distributed along the uterine layers, being up to 4 times higher on endometrium than myometrium 24 h after administration [53,57]. PpIX accumulation, induced by 5-ALA administration, seems to be selectively retained by the endometrium [54]. Indeed, higher PPIX was found in endometrial glands than in stroma [55,57]. A similar distribution was observed for BPD [56].

PDT outcome was evaluated using several photosensitisers. The photosensitisers were frequently administered *in uteri* solubilised in hyskon, a polysaccharide fluid used for uterine distention in hysteroscopy [55,56,59,62]. Bhatta and colleagues showed that HPD (Photofrin II^®^) doses of 1–2 mg/kg exposed to 100 J/cm^2^ were appropriate for endometrial ablation, resulting in substantial haemorrhage and cell death 24 h later. Necrosis was frequently observed [53,61]. Another study corroborated endometrial destruction after HPD-PDT (Photofrin^®^), showing decreased implantations 72 h after treatment with 0.7 mg/kg. Throughout the follow-up, atrophy was detected in treated horns, and no animals showed skin photosensitivity [58]. Benzoporphyn derivative (BPD) and tin ethyl etiopurpurin (SnET2) were also used [59,61].

Several studies reported 5-ALA effectiveness in endometrial ablation [54,56,57,59,61]. Uterine horns completely devoid of endometrium were achieved [54]. Endometrial outcomes varied with light fluency. Cases of minimal re-epithelisation were occasionally observed due to uneven light dosimetry [55]. Fehr and colleagues established an intensity of 64 J/cm^2^ to induce irreversible endometrial destruction [59]. Van Vugt and colleagues evaluated endometrial ablation in primates treated with 5-ALA. Moderate to complete endometrial ablation was observed at 1–2 cm depth. In menopausal animals, endometrial ablation was almost complete [63].

Following endometrial ablation, reproductive performance was frequently assessed by evaluating the implantation rate. After the 5-ALA-based photodynamic ablation implementation rate was significantly decreased [54,59]. Steiner and colleagues corroborated the lower number of implantations in the treated patients. When HPD based PDT was performed, limited reproductive capacity was observed [57,58].

Endometrial regeneration after PDT-induced endometrial ablation was described by Wyss and collaborators [64]. To evaluate the morphological changes, light and scanning electron microscopy studies were performed at 24 and 72 h and seven days after 5-ALA-PDT and 28 days after both 5-ALA- and BPD-PDT. Activation of the endometrium regeneration was observed 24 h after photodynamic ablation with 5-ALA, being completed 72 h later. Furthermore, proliferation was initiated in deeper glandular areas [64].

The literature reported three clinical studies using PDT for endometrial ablation, documented in Table 3. Wyss et al. performed PDT in three patients (two pre-menopausal and one postmenopausal) with intrauterine 5-ALA. Six months after treatment, patients reported a reduction in uterine bleeding. The microscopic analyses showed areas of thinned endometrial layers lacking glands [70]. The interval that leads to a maximal photosensitiser accumulation in the human endometrium and selectivity were critical points. The glandular selectivity was superior to the surrounding myometrium, and peak fluorescence was reached four to eight hours after photosensitiser administration. Other factors may influence mean fluorescence, namely menopausal state and endometrial cycle [70]. The same authors tested the 5-ALA intrauterine administration three to 152 days before hysterectomy. The approach resulted in necrosis and foci of preserved endometrium, despite no fibrosis, adhesions, or granulomatous reaction. A case of foreign-body giant cell reaction was described [71]. Necrosis was predominant in the acute phase of ablation, particularly in the first month, while healing and repair led to the recovery of endometrial thickness in the chronic phase. Atrophic glandless zones delineated by normal ou hyperplastic epithelium were described five weeks after PDT. Cornua and isthmus are main sources of regeneration. Residual glandular stumps and regeneration areas might be due to heterogeneous PpIX accumulation or light dosimetry [70,71], which is often a challenge.

The latest clinical trial included 11 patients median age of 42.3 years (35–52). PDT was performed using 5-ALA applied topically, reaching a short-term decrease in uterine bleeding [72]. Three months after treatment, endometrial regeneration occurred probably due to factors that can influence a complete and depth endometrial destruction. Also, the patients were under analgosedation, obviating the need for general or local anaesthesia [72]. Nevertheless, further research is needed to reach a long-term effect of PDT in endometrial ablation, namely developing more effective drugs and light delivery systems.

There is a preference of different photosensitisers for accumulation in endometrial tissues over surrounding tissues, which may be due to the constant renewal dynamics of rapidly dividing endometrial cells [73]. Additionally, the direct photosensitisers’ administration into the uterine cavity provides a minimally invasive modality to induce endometrial ablation. 

Several ex vivo studies evidenced the endometrial uptake and the distribution of the photosensitisers. In a series of studies, Gannon and colleagues investigated photosensitisers’ biodistribution. After 5-ALA topic administration, PpIX fluorescence extended to the deepest endometrial glands in the basal layer, which is essential because endometrial ablation depends on the destruction of the basal layer. PpIX concentration in the endometrium was nine to ten times higher than in the myometrium. However, the incomplete uptake through the endometrium layer may limit the clinical application [74]. Maximal fluorescence of the endometrial gland stumps is observed 4 to 8 h after photosensitiser administration, pointing to the optimised DLI to achieve endometrial ablation [75].

Maximum endometrial drug uptake and distribution of the benzoporphyrin were also investigated. The benzoporphyrin derivative monoacid ring A-induced fluorescence was maximum one hour after administration, with significantly higher uptake in endometrial glands than in the underlying stroma. No systemic drug uptake, side effects, or major technical difficulties were detected. Limited drug penetration and selective uptake by endometrial glands provided high safety for endometrial ablation [77].

A relevant aspect regarding PDT-induced endometrial ablation in light delivery. A device with three flexible optical fibres was tested on three human uteri. Homogeneous light distribution was achieved in the endometrial layer, while the power was less than 10% in the myometrium [78]. Postmenopausal ex vivo uteri showed a significantly lower light penetration depth than pre-menopausal in 14 human uteri. In normal-sized pre-menopausal uterine cavities, three diffusing fibres will deliver an optical dose above the photodynamic threshold level at a depth of 4 mm, even in the most remote areas [59]. Thus, the endometrium is an ideal organ for PDT since the endometrium is superficial, and the myometrium is a thick layer [78].

Thus, despite the promising results of inducing endometrial ablation with 5-ALA, a photosensitiser widely used in PDT-induced endometrial ablation studies, some in vivo and clinical studies showed endometrial regeneration after 5-ALA-PDT. These results encourage further investigation into novel photodynamic agents capable of inducing an effective and long-lasting action on the endometrium.

## 4. Future Perspectives and Recommendations

PDT presents significant advantages; it is a highly selective, local, and minimally invasive therapy. The studies reviewed in this manuscript point to some encouraging results. Nevertheless, clinical outcomes were often modest or unsatisfactory. Applications of PDT to endometrial disease were based on the primary photosensitisers, the majority developed with the advent of PDT. The vast majority of photosensitisers used in the studies included in this review have hydrophobic properties, limiting the therapeutic effect of PDT. Recently, different approaches have been explored to overcome low solubility and bioavailability and lack of tumour selectivity through the development of drug delivery systems [8,79,80]. Polymeric, lipidic and gold nanoparticles and liposomes are some of the drug delivery systems available that can be used to overcome the lack of solubility [8]. Beyond the ability to improve the photosensitiser solubility, block copolymer nanocarriers also present the capability to ameliorate biodistribution and pharmacokinetics [79]. Moreover, tumour targeting strategies can improve the photosensitiser’s selectivity, limiting the photosensitivity [8].

A low level of ROS at the tumour site is one of the major concerns regarding the efficacy of PDT as an anticancer modality. Thus, several oxygen producing or reducing dependence strategies have been investigated to surpass tumour hypoxia [81]. To increase singlet oxygen levels at the tumour area, self-quenching and Förster resonance energy transfer (FRET) quenching are commonly used in block copolymer nanocarriers [79]. A recent approach uses nanomaterials to produce oxygen or convert ROS into oxygen, such as catalase and peroxidase mimetic nanozymes [82].

Similarly, a decrease in the light potency during tissue penetration to reach deep tumours is a limitation of PDT, resulting in loss of efficacy. Different light delivery approaches, bioluminescence, or electromagnetic radiation-based strategies have been investigated to provide appropriate energy to activate photosensitisers into deep tissues. Moreover, indirect approaches were also explored, namely PDT-induced immune stimulation or combined modalities [83].

Several new developments and photosensitisers with ameliorated properties are available or under research [5,84]. Thus, a primordial question remains: might new photosensitisers offer better outcomes for endometrial disease? 

For instance, a novel type 4,5,6,7-tetrahydropyrazolo [1,5-*a*]pyridine-fused chlorins bear outstanding characteristics: high absorption in the 600–800 nm spectral region, which is ideal for PDT because red wavelengths go deeper into the tissues; high phototoxicity in very low concentrations (nanomolar range); and, proved potency in vivo, namely in chick chorioallantoic membrane and mice [39,85,86,87]. Another fascinating feature of the ring-fused chlorins is the possibility of structural modulation while retaining photophysical and photochemical features. In a single molecule, the platinum derivatives offer the possibility of PDT and diagnosis of the tumour by luminescence imaging [39,88]. In fact, PDT based theranostics could be a valuable approach to endometrial disease.

Conservative treatment in endometrial cancer could result from combined multimodal therapies, with a hysteroscopic approach, eradicating the potentially remaining tumour by PDT, and suppressing recurrence by adjuvant medical treatment. In general, photosensitisers reach higher concentrations in tumour tissue compared with the adjacent normal tissue. These phenomena improve functional outcomes because of the negligible effects on normal functional structures, which is a major point in fertility-sparing. Additionally, higher concentrations in tumour cells also conveys photosensitisers the possibility of being used as photodiagnosis agents. The identification of cancer cells could guide surgical resections [89], similar to what is currently approved for gliomas and bladder cancer [90,91,92,93]. Photodiagnosis has been applied to gynaecologic diseases [10]; however, scarce studies were found regarding endometrial diseases, 5-ALA being the only photosensitiser used [65,94,95,96].

Another question to be addressed is the outcomes of PDT in endometrial cancer stem cells (CSC). Endometrial CSC are responsible for tumourigenesis, resistance to treatment and recurrence [97]. This population can be identified by surface markers, enzymatic activity and a functional profile, being easily isolated from cell lines and primary tumour samples [98]. The perspective of using PDT to eradicate these cells could provide a practical, conservative and recurrence-free approach, particularly valuable for fertility-sparing, patients with surgical risk and recurrent disease.

The application of PDT to benign endometrial disease seems safe and selective. Prior investigations systematised in this review have pointed out the preferential accumulation of photosensitisers in endometrial lesions. PDT could offer an effective local approach, with limited side effects, particularly for recurrent diseases. In this application, we expect better outcomes with new photosensitisers designed for topical application [99,100].

## 5. Conclusions

Research regarding PDT in endometrial pathology used mostly clinically approved photosensitisers. Using continuous cell lines and primary samples, the outcome was evaluated on in vitro models of cancer, adenomyosis and endometrioses, while most animal studies focused on endometrial ablation. A few clinical attempts used PDT for endometrial ablation and cancer lesions. This review emphasises a promising field of research in the conservative treatment of endometrial benign and malignant lesions, particularly focusing on the improved results that can be achieved with new photosensitisers.

## Figures and Tables

**Table 1:** In vitro studies.

Ref.	Disease, Model	PDT	Methods	Main Results
[21]	Endometrial carcinoma, HEC1-1A cell line	**PS**: HPD, 1.25–80 µg/mL **DLI**: 12–72 h **Light**: 630 nm 40–100 mW/cm^2^, up to 20 J/cm^2^ **NT**: single	Cell viability	The photosensitiser *per se* did not cause any changes. PDT led to the loss of viability, with complete cell death for the more potent treatments.
[23]	Cervical or endometrial cancer, Hela cell line	**PS**: HPD (Photofrin), 20 µM **DLI**: 3 h **Light**: 2.5 mW/cm^2^ 630 nm, up to 3.3 mJ **NT**: single	ROS levels assessment (fluorimetry); Apoptosis/necrosis assay (confocal microscopy); MTT assay	While hydroxyl radical and superoxide anion concentrations remained stable after PDT, a slight increase in hydrogen peroxide was observed. When PDT was combined with carboplatin, ROS concentrations raised. PDT increased necrotic cells while PDT combined with carboplatin increased apoptotic and necrotic cells. The viability of cells submitted to PDT was dependent on the light energy deposited. Association with carboplatin showed better outcomes when low light energies (330–660 mJ) were used.
[25]	Endometrial cancer; primary cells, 10 cases	**PS**: HPD, 0.1 mg/L **DLI**: 24 h **Light**: 620–640 nm 18 J/cm^2^ 75 mW **NT**: single	Immunohistochemistry (H&E, laminin, EGFR, nucleolar organised regions staining)	PDT induced no damage to the basal membrane (laminin) but decreased EGFR and proliferation, while enhancement of necrosis was observed.
[24]	Endometrial carcinoma, HEC-1B cell line	**PS**: Hypericin, 0.15 µM **DLI**: 16 h **Light**: 599 nm 2–10 J/cm^2^; 599 nm 2 + 5 J/cm^2^ spaced 3 or 20 h **NT**: Single or double irradiation spaced of 3 or 20 h	Cell photosensitisation; Cell uptake; Cell cycle (FACS); Western blot	PARP activation determined apoptotic cell death, while necrosis was observed for higher fluences. Hypericin uptake remained quite steady from 3 to 20 h. Sub-cellular fractions showed a tendency for nuclear accumulation. Photoactivation stimulated HSP70 synthesis, P21 and P53 expression. The cell cycle seemed to accumulate in the G2/M phase.
[20]	Endometriosis; primary epithelial cells from endometriotic foci, 15 cases	**PS**: 5-ALA, 1–8 mM **DLI**: 2 and 4 h **Light**: 30 mW 56 J/cm^2^ 635 nm; electric bulb (75 W) **NT**: single	PpIX uptake; Cell death; Rhodamine 123 staining	Accumulation of PpIX was mostly noted after two hours of 5-ALA incubation. Laser irradiation resulted in gradually rising apoptotic cells.
[22]	Endometrial carcinoma, HEC1-1A cell line	**PS**: 5-ALA, 0.5 mM; hypericin, 60 nM; 5-ALA + hypericin **DLI**: 4 h **Light**: 2.5 J/cm^2^ 635 nm or 400–800 nm **NT**: single	Clonogenic assay; HPLC PpIX quantification	5-ALA PDT decreased cell survival. No enhancement was seen by combining 5-ALA with hypericin after 635 nM irradiation. A sight additive effect was observed if irradiation was made with white light. 5-ALA was superior to hypericin in the conditions tested.
[30]	Endometriosis; primary epithelial cells from eutopic (normal) and ectopic endometria, 8 and 15 cases, respectively	**PS**: 5-ALA, 1–8 mM **DLI**: 2 h **Light**: 56 J/cm^2^ 635 nm **NT**: single	Blocking P-GP using verapamil; XTT Assay; Immunohistochemistry	Endometriotic cells were significantly more responsive to PDT than normal endometrium. PDT caused significant cell growth inhibition, which was potentiated by association with a PGP inhibitor (verapamil).
[31]	Endometriosis; primary epithelial cells from eutopic (normal) and ectopic endometria, 15 cases each	**PS**: 5-ALA (PpIX), 2.0 mM **DLI**: 2 h **Light**: 30 mW/cm^2^ 635 nm **NT**: single	PpIX uptake (confocal microscopy); XTT assay	Normal and endometriotic cells accumulate PpIX, which increases after hormonal stimulation. Endometriotic cells were significantly more responsive to PDT than normal endometrium.
[32]	Adenomyosis; primary cells from adenomyosis and endometrium	**PS**: 5-ALA, 100 mg/mL **DLI**: 8, 24 and 48 h **Light**: 635 nm **NT**: single	Immunocytochemistry; MTT assay	Loss of cell viability, dependent on the 5-ALA incubation time and irradiation. Adenomyosis cells were more susceptible to PDT than stromal cells.
[16]	Endometrial carcinoma, HEC-1-A cell line	**PS**: Radachlorin, 2.5–200 µM **DLI**: 4 h **Light**: 50 mW 660 nm, until 25 J/cm^2^ **NT**: single	Morphology (microscopy); MTT assay; TUNEL assay, Tube formation assay; Invasion assay; Prostaglandin E2 assay; Western Blot	PDT treated cells showed condensed cytoplasm or floating growth patterns. IC_50_ of 55.4 µM and 20 µM were obtained 24 and 48 h after PDT, respectively. Annexin-V-positive and TUNEL-positive cells increased after PDT treatment, mostly after 48 h. PARP and caspase 9 increased when PDT was combined with VEGF treatment. PDT reduced tubular formation, and PDT + VEGF conditions led to more robust inhibition of tubular formation. PDT (and PDT combined with VEGF) suppressed the invasion. PDT (and PDT combined with VEGF) reduced prostaglandin 2 production. PDT combined with VEGF reduced EGFR, VEGFR2 and RhoA expression.

Abbreviations: DLI, drug-light interval; HPD, hematoporphyrin derivative; NT, number of treatments; PS, photosensitiser.

**Table 2:** In vivo studies.

Ref.	Disease/Intervention	Species	PDT	Methodology	Main Results
[46]	Endometriosis	**Sp.**: New Zealand white rabbits **Md.**: Peritoneal autotransplantation of endometrial tissu **n.**: 10 rabbits	**PS**: HPD, *iv.*, 50 mg/kg **DLI**: 24 h **Light**: *iu.*, 627.8 nM, 0.5–1.3 W/cm^2^, up to 100 J/cm^2^	Bioavailability (fluorescence); Histopathology	Endometriotic lesions show higher HPD fluorescence. PDT lead to tissue damage, namely necrosis and hyperaemia, with preservation of the surrounding tissues.
[47]	Endometriosis	**Sp.**: New Zealand white rabbits **Md.**: Peritoneal autotransplantation of endometrial tissue **n.**: 15 rabbits (virgin)	**PS**: HPD, *iv.*, 10 mg/kg **DLI**: 24 h **Light**: *iu.*, 630 nm, 100–210 mW/cm^2^; up to 50 or 100 J/cm^2^	Histopathology	81% and 60% of the transplants treated with 100 J/cm^2^ and 50 J/cm^2^ presented complete or almost complete endometrial epithelial destruction, respectively.
[48]	Endometriosis	**Sp.**: Sprague-Dawley rats **Md.**: Peritoneal autotransplantation of endometrial tissue **n.**: 38 rats (mature)	**PS**: 5-ALA, *iv.*, 100 and 400 mg/kg **DLI**: 3 h **Light**: *ip.* (surgical), 600–700 nm 340 ± 20 mW; 5–15 min	Histopathology	Endometrial ablation of all explants collected 3 to 4 days after PDT, and absence of regrowth after three weeks. Peritoneal necrosis recovered after 16 days.
[32]	Adenomyosis	**Sp.**: Immunodeficient nude mice **Md.**: Transplantation of human adenomyosis tissues in ovariectomised animals. (Oestradiol transdermal administration.) **n.**: 8 6–8 weeks mice (mature)	**PS**: 5-ALA, *ip.*, 0.8 g/kg **DLI**: 3 h **Light**: *ip.*, 630 nm, 100 mW	Bioavailability (fluorescence); Histopathology	Higher fluorescence was detected in adenomyosis tissues than in myometrium, with a maximum 3 h after administration. A decrease in the number of epithelial and particularly stromal cells was observed in adenomyosis tissues after PDT. No necrotic cells were observed.
[53]	Endometrial ablation	**Sp.**: New Zealand white rabbits **Md.**: n.a. **n.**: 58 rabbits (virgin)	**PS**: Photofrin II, *iv.*, 0–10 mg/kg **DLI**: 4, 24 and 48 h **Light**: *iu.*, 630 nm, 100–200 J/cm^2^	Bioavailability (fluorescence and microscopy); Histopathology	Bioavailability in the endometrium was up to 4 times higher than in the myometrium. For endometrial ablation, PS doses of 1–2 mg/kg and 100 J/cm^2^ showed to be appropriate, with preservation of surrounding organs. PDT resulted in extensive haemorrhage and cell death 24 h after treatment and necrosis five days later.
[54]	Endometrial ablation	**Sp.**: Sprague-Dawley rats **Md.**: n.a. **n.**: 51 + 24 rats (mature)	**PS**: 5-ALA, *iu.*, 4–16 mg **DLI**: 3 h **Light**: *iu.*, red light, 150 J/cm^2^, 30 min	Short- and long-term outcomes (10 and 60 days); Implantation rate; Histopathology	PDT ablation decreased implantation rate and endometrial atrophy.
[55]	Endometrial ablation	**Sp.**: New Zealand white rabbits **Md.**: n.a. **n.**: 18 rabbits (mature)	**PS**: BDP, *iu.*, 2 mg **DLI**: 1.5 h **Light**: *iu.*, 690 nm, 195 mW, 40–80 J/cm^2^	Bioavailability (fluorescence); Histopathology (optical and SEM)	Glandular fluorescence was superior to stroma and myometrium. The maximum fluorescence was noticed 1.5 h after administration. The histological study showed endometrial epithelium destruction after treatment with minimal regeneration.
[56]	Endometrial ablation	**Sp.**: Sprague-Dawley rats and New Zealand white rabbits **Md.**: *n.a.* **n.**: 12 rats (mature) and 42 rabbits (mature)	**PS**: 5-ALA, *iu.*, up to 480 mg **DLI**: 3 h **Light**: *iu.*, 630 nm 255 mW, 80–160 J/cm^2^	Bioavailability (fluorescence); Histopathology (optical and SEM)	The maximum fluorescence of protoporphyrin IX was reached 3 h after administration, proving higher in the glands. Epithelial destruction was observed in the histological studies, revealing a low regeneration.
[57]	Endometrial ablation	**Sp.**: Sprague-Dawley rats **Md.**: n.a. **n.**: 87 rats (mature)	**PS**: 5-ALA, *iu.*, 58 mg/kg **DLI**: 3 h **Light**: *iu.*, 630 nm, 133 mW/cm^2^ up to 212 J/cm^2^ (100 J/cm^2^ for the photosensitivity study)	Bioavailability (fluorescence); Implantation rate; Skin photosensitivity; Thermogenic effect; Histopathology	The fluorescence study revealed a higher photosensitiser concentration in the glands 3 to 6 h after administration. Endometrial destruction with atrophy was observed 7 to 10 weeks after PDT. The number of implantations was significantly lower in treated horns.
[58]	Endometrial ablation	**Sp.**: Sprague-Dawley rats **Md.**: n.a. **n.**: 74 rats (mature)	**PS**: HPD (Photofrin), *iu.*, 0.7 mg/kg **DLI**: 3, 24 and 72 h **Light**: *iu.*, 630 nm, 100 mW/cm^2^ up to 80 J/cm^2^ (200 mW/cm^2^ up to 100 J/cm^2^ for the photosensitivity study)	Bioavailability (fluorescence); Implantation rate; Skin photosensitivity	Photofrin diffusely distributed along the endometrium and myometrium. A significant reduction in implantations was observed in PDT treated horns. No skin photosensitivity was noticed.
[59]	Endometrial ablation	**Sp.**: Sprague-Dawley rats **Md.**: *n.a.* **n.**: 125 rats (mature)	**PS**: 5-ALA, *iu.*, 30 mg **DLI**: 3 h **Light**: *iu.*, 630 nm, 100 mW/cm^2^; ranging 8–160 J/cm^2^	Histopathology; Thermogenic effect; Implantation rate	Temperatures never exceeded 40 °C during PDT. Endometrial outcomes varied with light fluency. The deposition of 43 J/cm^2^ led to endometrial stroma and myometrium damage with regeneration within three weeks. Higher intensity of 64 J/cm^2^ determined irreversible endometrial destruction. A reduced number of implantations was observed after PDT.
[60]	Endometrial ablation	**Sp.**: Sprague-Dawley rats **Md.**: n.a. **n.**: 32 rats	**PS**: BPD, *iv.*, 0.0625–2 mg/kg **DLI**: 5 min **Light**: *iu.*, 630 nm 100 mW/cm, until 120 J/cm^2^	Histopathology	Endometrial destruction was observed in all treatment groups. The most significant degree of destruction was obtained at the higher dose of 2 mg/kg. Gland destruction and myometrium conservation were achieved with doses of 0.5 to 0.0625 mg/kg.
[61]	Endometrial ablation	**Sp.**: Sprague-Dawley rats **Md.**: n.a. **n.**: 30 rats (mature)	**PS**: 5-ALA, *iu.*, 0.1 mL of 10 mg/mL solution **DLI**: 3 h **Light**: *iu.*, 600–700 nm, 280 ± 40 mW, 180 J/cm^2^ (10 min) or 630 nm, 300 mW, 1080 J/cm^2^ (60 min)	Histopathology; Thermogenic effect	PDT resulted in extensive histologic damage to all layers of the uterine wall. Specimens were devoid of luminal epithelium and endometrial glands, while stromal oedema was prominent. Moreover, damage to the circular myometrium and focal necrosis throughout the longitudinal outer layer of the myometrium was evident. During irradiation, a temperature rise to 46 °C was observed.
[62]	Endometrial ablation	**Sp.**: Sprague-Dawley rats **Md.**: n.a. **n.**: 45 rats (8–10 weeks)	**PS**: SnET2, *iv.* (tail vein) or *iu.*, 2 mg/kg (*iv.*) or 60 µg (*iu.*) **DLI**: 3 and 24 h **Light**: *iu.*, 665 nm, 100 mW/cm^2^, up to 200 J/cm^2^	Fluorescence detection; Histopathology	The fluorescence study revealed the highest levels of photosensitiser 3 h after administration. Effective endometrial ablation was observed through intrauterine administration of SnET2 at 150 J/cm^2^ with a DLI of 24 h. Necrosis extent was light-dose dependent.
[63]	Endometrial ablation	**Sp.**: Rhesus monkeys (and one cynomolgus monkey) **Md.**: *n.a.* **n.**: 18 + 1 monkeys, 5–20 years	**PS**: 5-ALA, *iu.*, 250 mg/mL **DLI**: 4 h **Light**: *iu.*, 635 nm 300 mW; 60 min (continuous or fractionated)	Histopathology; Thermogenic effect	Endometrial ablation was observed in all animals ranging from moderate to complete. The greatest degree was seen in menopausal monkeys. The luminal temperature increased up to 50 °C, whereas no significant increases were seen in light controls.
[64]	Endometrial ablation	**Sp.**: New Zealand white rabbits **Md.**: n.a. **n.**: 15 rabbits (mature)	**PS**: 5-ALA and BPD, *iu.*, ALA: 2.4 g; BPD: 24 mg **DLI**: ALA: 3 h; BPD: 1.5 h **Light**: *iu.*, ALA: 630 nm, 40–80 J/cm^2^; BPD: 690 nm, 40–80 J/cm^2^	Histopathology (optical and SEM)	Endometrium regeneration was activated 24 h and completed 72 h after photodynamic ablation. Proliferation initiated in deeper regions of the glands.

Abbreviations: BPD, benzoporphyrin derivative; DLI, drug-light interval; HPD, hematoporphyrin derivative; *ip.* intraperitoneal; *iu.*, intrauterine (laparotomy); *iv.*, intravenous; Md., model; n., number; PS, photosensitiser; Sp., species; SnET2, tin ethyl etiopurpurin.

**Table 3:** Clinical studies.

Ref.	Disease	N	Age	PDT	Follow Up	Results
[27]	Endometrial carcinoma, metastatic	1	*n.d.*	**PS**: HPD, 5 mg/kg, *iv.* **DLI**: *unclear* **Light**: 600–700 nM, 100 mW/cm^2^	*n.d.*	Complete response in six tumour sites.
[28]	Endometrial adenocarcinoma, recurrent	5	67.5 years (median age)	**PS**: HPD, 5 mg/kg, *iv.* **DLI**: 48 h **Light**: 630 (and 514 nm in some cases), 60–500 J/cm^2^	Up to 92 months	In the cases where PDT was used as palliative, absence of symptoms for at least 60 days was observed in 66,6% of the cases. In the curative intent, PDT complete response was achieved in 70,8%. Survival ranged from three to 92 months. Obs.: Avoidance of direct sunlight for 30 days.
[29]	Endometrial carcinoma, stage 1a	7	60–81 years	**PS**: HPD, 2 mg/kg **DLI**: 24–72 h **Light**: 632 nm, 200 J/cm^2^	12 months	Despite five out of seven cases of initial (1 month) complete response, after one year, four patients relapsed. Obs.: Avoidance of direct sunlight for 38 days.
[76]	Endometrioid adenocarcinoma, grade 1 or 2	16	24–35 years	**PS**: HPD, 2 mg/kg, *iv.* **DLI**: 48 h **Light**: 630 nm, 600 mW, 900 seg	Up to 140 months	Complete response in 12 cases (75%) and 4 cases of recurrence. Among seven women who attempted to get pregnant, four had successful pregnancies. Adverse effects: four cases of reversible mild facial angioedema
[41]	Endometrial sarcoma, low grade	1	31 years	**PS**: HPD, 3 mg/kg, *iv.* **DLI**: 48 h **Light**: 631 nm, 600 mW, 900 seg	99 months	No evidence of recurrence during 99 months. After 32 months, an IVF was successful with the delivery of twins. Obs.: Adjuvant treatment with an aromatase inhibitor.
[70]	Abnormal uterine bleeding	3	*n.d.*	**PS**: 5-ALA, 1.5 mL, 400 mg/mL solution, *iu.* **DLI**: 4–6 h **Light**: 635 nm, 160 J/cm^2^	Six months	Reduction of uterine bleeding. Normal endometrium and thinned endometrial layers lacking glands, evidence of photodynamic destruction limited to endometrial layers in a case submitted to hysterectomy.
[71]	Abnormal uterine bleeding	4	>30 years	**PS**: 5-ALA, 1.5–2 mL, 100–400 mg/mL solution, *iu.* **DLI**: 4–6 h **Light**: 635 nm, 160 J/cm^2^	At least 152 days	Necrosis was found three days after PDT. Foci of preserved endometrium, no fibrosis or adhesions in all patients. Obs.: Hysterectomy performed 3, 35, 92 and 152 days after PDT.
[72]	Abnormal uterine bleeding	11	42.3 years (median age)	**PS**: 5-ALA, *iu.* **DLI**: 3–6 h **Light**: 636 nm, 160 J/cm^2^	Six months	There was a significant reduction in menstrual blood loss up to three months after treatment. Later on, the PDT effect was less obvious Obs.: PDT repetition in four cases

Abbreviations: DLI, drug-light interval; HPD, hematoporphyrin derivative; *iu.*, intrauterine (instilation): *iv.*, intravenous; *n.d.*, not disclosed.

## Data Availability

Data are contained within the article.
